# Dietary Intake of Folate, B-Vitamins and Methionine and Breast Cancer Risk among Hispanic and Non-Hispanic White Women

**DOI:** 10.1371/journal.pone.0054495

**Published:** 2013-02-11

**Authors:** Dongyan Yang, Richard N. Baumgartner, Martha L. Slattery, Chenxi Wang, Anna R. Giuliano, Maureen A. Murtaugh, Betsy C. Risendal, Tim Byers, Kathy B. Baumgartner

**Affiliations:** 1 Department of Epidemiology and Population Health, School of Public Health and Information Sciences, University of Louisville, Louisville, Kentucky, United States of America; 2 Department of Medicine, University of Utah, Salt Lake City, Utah, United States of America; 3 Moffitt Cancer Center and Research Institute, Tampa, Florida, United States of America; 4 University of Colorado School of Public Health, Denver, Colorado, United States of America; Dartmouth, United States of America

## Abstract

**Background:**

Low dietary folate intake is associated with several neoplasias, but reports are inconsistent for breast cancer. Additionally, the association of folate with breast cancer estrogen receptor (ER) status is not well established.

**Objective:**

To determine if dietary intakes of folate, B-vitamins (B_2_, B_6_, B_12_) and methionine are associated with breast cancer risk and ER status in Hispanic, and non-Hispanic White women in the southwestern US.

**Materials and Methods:**

Primary breast cancer cases (n = 2,325) in the 4-Corners region (Arizona, Colorado, New Mexico and Utah), diagnosed between October 1999 and May 2004, were identified through state cancer registries. Controls (n = 2,525) were frequency-matched by ethnicity and age (±5 years). Dietary intake, physical activity and other exposures were assessed using in-person interviews. Risk was assessed through multivariable and multinomial logistic regression with adjustment for relevant covariates.

**Result:**

While there was no overall association with breast cancer, the highest quartile of folate intake was marginally inversely associated with ER− breast cancer (Odds Ratio (OR) = 0.50, 95%CI 0.25–1.00, p for trend = 0.07). Vitamin B_12_ intake was inversely associated with breast cancer also (OR = 0.73, 95%CI 0.53–1.00, p for trend = 0.06), particularly for the highest quartile of ER+ breast cancer (OR = 0.67, 95%CI 0.46–0.99, p for trend = 0.06), among NHW women (OR = 0.49, 95%CI 0.29–0.81, p for trend = 0.01) and invasive breast cancer (OR = 0.63; 95%CI: 0.42, 0.93, *P*_trend_ = 0.01). Methionine intake was also inversely associated with ER+ breast cancer (OR for 4^th^ quartile = 0.83, 95%CI 0.66–1.03, p for trend = 0.04), primarily among Hispanic women (OR = 0.71, 95%CI 0.47–1.06, and P for trend = 0.02).

**Conclusion:**

Higher intake of folate is marginally associated with a lower risk for ER− breast cancer, and higher intakes of vitamin B-12 and methionine are marginally associated with a lower risk of ER+ breast cancer.

## Introduction

Dietary folate, methionine, vitamins B6 and B12 participate in one-carbon metabolism, which is essential for DNA methylation, synthesis and repair [1]. Low dietary folate intake has been associated with several neoplasias [2], [3], [4], [5], [6], [7]. A meta-analysis of 13 case-control studies [8] reported a significant, protective summary effect of folate intake on breast cancer risk (odds ratio (OR) = 0.73; 95% confidence interval (CI): 0.64, 0.83). Evidence from cohort studies, however, is neither consistent nor conclusive. Two recent cohort studies reported an inverse association [9], [10], and some studies have indicated that the reduced risk may be more apparent in certain populations, e.g. women with high levels of alcohol intake [11], [12], [13], [14] or premenopausal Chinese women. [15]. Reported associations with methionine or vitamins B6 and B12 generally have been null [16].

The majority of women have estrogen receptor (ER) positive tumors and respond to hormone therapy [17], [18], [19], but approximately one-third of women have ER− tumors that are refractory to hormonal treatment and associated with a poor outcome. Hispanic and African American women are more likely to develop ER− tumors [17], [20], [21]. Whether folate intake affects ER expression status is not well established. Maruti et al. [9] reported that long-term, average folate intake had a significant, protective association with ER−, but not ER+, breast cancer; current intake, however, showed no association. Another recent cohort study reported a non-significant trend towards a protective association with ER− breast cancer [15]. Theoretically, the association of ER− status with folate intake and other B vitamins could be due to methylation of the CpG island promoter region for the ER gene [22] and several studies have detected this association [10], [23], [24].

We investigated the association of dietary intake of folate, B-vitamins (B_2_, B_6_, B_12_), and methionine with breast cancer risk and ER status among Hispanic and non-Hispanic White (NHW) women using data from the 4-Corners Breast Cancer Study (4-CBCS), a multi-centered, population-based case-control study [25], [26]. Our primary hypothesis was that folate intake is inversely associated with breast cancer risk, specifically the ER− phenotype, and that inverse associations would be present for the other B-vitamins (B_2_, B_6_, B_12_) and methionine. Additionally, we evaluated the potential modification of these associations by menopausal status, ethnicity, and alcohol intake.

## Materials and Methods

### Study Population

Participants in the 4-CBCS were women between 25 and 79 years of age residing in Arizona, Colorado, New Mexico and Utah. Subject selection methods, participation rates, data collection, and quality control procedures are described elsewhere [26], [27], [28], [29]. Cases diagnosed with a new primary breast cancer, *in situ* or invasive disease, with a histological confirmation between 10/1999–05/2004 were ascertained through state cancer registries. All Hispanic and non-reservation American Indian cases were included. A five-year age-matched sample of non-Hispanic white cases was selected randomly on a 1∶1 ratio to the distribution of Hispanic/American Indian cases in Arizona and Colorado and at a 4∶1 ratio in Utah. In New Mexico, all Hispanic/American Indian and non-Hispanic white cases under age 50 were included with a 1∶1 ratio for women over 50 years. Controls <65 were selected randomly from commercial mailing lists (Arizona, Colorado) or driver’s license lists (New Mexico, Utah) and from Center for Medicare Services lists if > = 65 years, during the same time-period as cases, and were frequency-matched to cases by ethnicity and age within five years. The computer program, Generally Useful Ethnic Search System [30], [31] and the Census Spanish Surname List [32] were used to identify Hispanic women when ethnicity was unknown. Eligibility and ethnicity were confirmed at the time of the screening telephone contact. Contact was made with 71 percent of Hispanic/American Indian women (75% cases; 66% controls) and with 80 percent of non-Hispanic whites (85% cases; 75% controls). Participation rates for cases and controls based on all subjects contacted were 62 percent and 42 percent, respectively [28]. Twenty-four percent of participants were Hispanic/American Indian. All participants signed an informed written consent prior to participation. The study was approved by the Institutional Review Board for Human Subjects Research at each institution.

All participants were asked to complete an in-person interviewer-administered computerized questionnaire in English or Spanish, based on the participant’s preference, blood draw, and anthropometric measurements. Data on demographic characteristics and breast cancer risk factors prior to the referent year (year prior to diagnosis date for cases; selection date for controls) were collected, including reproductive, medical and medication history, height, weight history, diet, physical activity, education, cigarette smoking, and alcohol consumption. There were few American Indian participants (0.85%) and their data were combined with Hispanic women in analyses. Findings based on analyses by genetic admixture supports this combined grouping [33].

#### Folic acid

A diet history questionnaire [34], adapted from the Coronary Artery Risk Development in Young Adults questionnaire [35] to include the current food supply and ethnic foods commonly consumed in the southwestern United States [29], was used to collect data for frequency (daily, weekly, monthly), serving amount, type of preparation, usual serving size, and type of fat used for food preparation. Intake of energy and over 120 nutrients was calculated using the Nutrition Data System for Research (Database Version, 4.02_30_ Regents of the University of Minnesota). Folate intake was calculated with and without multivitamin supplementation as well as intake pre- and post-fortification.

#### Potential confounders

Physical activity was measured using a modified version of the Cross Cultural Activity Participation Survey [36] and expressed as total metabolic equivalent (MET) values for moderate and vigorous activities during the referent year [37]. Body mass index was calculated as weight in kilograms (kg)/height in square meters (m^2^) for the referent year. Menopausal status was determined by an algorithm based on age (<57 vs. > = 57 years) at referent date and responses to eight questions regarding menstrual status, hormone replacement use, and surgical or medical menopause [27]. The peri-menopausal category which included 561 (12%) women was combined with the pre-menopausal group for analysis. Cigarette smoking status was categorized as never, former, or current smoker. Alcohol consumption for referent year was categorized as low (0 − <10g/day) vs. moderate (> = 10g/day) consumption. Parity was categorized as: nulliparous; 1–2 live births; 3–4 live births; and 5 or more live births. Family history of breast cancer was based on report for first degree relatives. Recent oral contraceptive use was defined as self-reported use within five years prior to referent date. Education was defined as high school or less vs. some college. Data on clinical and tumor characteristics, including ER status, were obtained from cancer registry data.

### Data Analysis

Separate calculations were made for dietary folate based on pre- and post-fortification, which was initiated in 1998 [38]. Mean intakes of nutrients of vitamins B_2_, B_6_, B_12_, and methionine were also calculated. Cases and controls were compared by ethnicity, as well as the two ethnicities. All the differences were evaluated by least-squares regression analysis of variance, adjusting for age, center and total energy intake. Groups were compared for covariates using *t* tests for continuous variables and chi-square tests for categorical variables. Unconditional logistic regression models were used to estimate the odds ratios, and 95% confidence intervals for associations between folate, vitamins B_2_, B_6_, B_12_ and methionine with breast cancer risk. Nutrient intakes were log-transformed and adjusted for total energy intake using the residual method [39], [40], and then categorized based on the quartile distribution of the residuals in controls, consistent with previous studies [9], [41], [42] The lowest quartile was the referent category in all models and the order of each category was used as a continuous term to test for linear trend across categories using the Wald statistic to test the resulting coefficient [43]. Analyses were conducted based on diet only, and on total intake (dietary nutrient plus supplement); however, results are provided for only total intake as there were no meaningful differences between the analyses. Although we evaluated both pre and post-fortification, only pre-fortification analyses are reported since relevant exposure occurred prior to the introduction of fortification.

Analyses were based upon two models: (1) folate, vitamins B_2_, B_6_, B_12_ and methionine adjusted for center, age, and ethnicity; and (2) additional adjustment for education, BMI, energy intake/day, fiber intake/day, total weekly MET hours of physical activity, cigarette smoking status, recent alcohol consumption, parity, family history and oral contraceptive use five years prior to referent date. Although the impact of any one covariate was minimal (<15% for the 4^th^ folate quartile), all variables were retained in the final models for consistency with previous studies. Multinomial logistic regression was used to estimate risk by ER tumor status. Effect modification of folate (continuous) by menopausal status, ethnicity, and alcohol consumption was evaluated by comparing models with and without interaction terms using likelihood ratio test [43]. Stratified analyses by ethnicity and menopausal status were also performed. All p values were two-sided. SAS statistical software (version 9.13, SAS Institute, Cary, NC) was used to perform analyses.

### Ethics Statement

All participants signed an informed written consent prior to participation. The study was approved by the following Institutional Review Boards: The Human Research Protections Office of University of New Mexico; University of Utah Institutional Review Board; Human Subjects Research and Institutional Review Board of University of Arizona and Human Research and the Institutional Review Board of University of Colorado.

## Results

A total of 2,325 cases (H = 798; NHW = 1,527) and 2,525 controls (H = 924; NHW = 1,601) participated in the study. Of these, 152 subjects with extreme values for BMI (<16 kg/m2 or >50 kg/m2) or caloric intake (<600 kcal/day or >6500 kcal/day) were excluded, leaving a total of 2,262 cases (H = 765; NHW = 1,497) and 2,436 controls (H = 877; NHW = 1,559). ER status was present for 65% of all cases; 17% for women diagnosed with *in situ* vs. 82% for women with invasive cancer. Analyses based on ER status therefore included a total of 1,480 cases. Hispanic cases were significantly more likely to have ER− tumors than non-Hispanic white cases (27% vs. 20%, p = 0.003). The average age of the participants was 55 (±12) y.

There were statistically significant differences between ethnic groups for all covariates, regardless of case-control status, with the exception of oral contraceptive use (Table 1), many of which have been previously reported [26], [27], [44], [45], [46], [47], [48], [49], [50], [51], [52], [53], [54]. Hispanic women reported more live births, a higher total energy intake, a lower number of MET hours per week, a higher prevalence of overweight and obesity, a lower prevalence of cigarette smoking, and lower levels of alcohol consumption compared to NHW women. Hispanic women were diagnosed more frequently with regional breast cancer compared with NHW women.

**Table 1:** Characteristics of breast cancer cases and controls, stratified by ethnicity, 4-Corners Breast Cancer Study, 1999–2004.

	Non-Hispanic White					Hispanic					
	Case		Control		p value^a^	Case		Control		p value^a^	p value b
	No.	%	No.	%		No.	%	No.	%		
Subjects	1,497	49	1,559	51		765	46.52	877	53.48		
Estrogen receptor tumor status											0.03
ER−	197	19.98	–	–		133	26.98	–	–		
ER+	789	80.02	–	–		361	73.02	–	–		
Age (years)					<0.01					<0.01	<0.01
< = 30	11	0.73	22	1.41		11	1.44	17	1.94		
30–40	110	7.34	113	7.25		104	13.63	86	9.81		
41–50	446	29.77	423	27.13		247	32.37	254	28.96		
51–60	448	29.91	402	25.79		218	28.57	225	25.66		
61–70	330	22.03	360	23.09		133	17.43	204	23.26		
>70	153	10.21	239	15.33		50	6.55	91	10.38		
Education level^c^					0.68					0.53	<0.01
High school or less	363	24.23	401	25.72		436	57.14	486	55.42		
Some college	552	36.85	582	37.33		212	27.79	244	27.82		
College Degree or more	582	38.85	575	36.88		111	14.55	145	16.53		
Family history, 1^st^ degree^d^					<0.01					0.04	<0.01
Yes	333	22.53	221	14.4		123	16.42	110	12.82		
No	1145	77.47	1314	85.6		626	83.58	748	87.18		
Body mass index					0.11					0.04	<0.01
<25	719	48.00	714	45.8		259	33.94	248	28.28		
25–30	441	29.44	443	28.42		264	34.60	318	36.26		
30+	338	22.56	402	25.79		240	31.45	311	35.46		
Cigarette smoking status^e^					0.08					0.92	<0.01
Current	202	13.49	196	12.6		90	11.8	109	12.46		
Former	471	31.46	441	28.36		169	22.15	191	21.83		
Never	824	55.04	918	59.04		504	66.06	575	65.71		
Alcohol consumption					0.11					0.16	<0.01
Low (0−<10 gm/day)	1236	82.51	1320	84.67		708	92.79	797	90.88		
High (> = 10 gm/day)	262	17.49	239	15.33		55	7.21	80	9.12		
Parity					<0.01					<0.01	<0.01
Nulliparous	254	16.96	220	14.11		75	9.83	86	9.81		
1 to 2	684	45.66	643	41.24		314	41.15	292	33.3		
3 to 4	468	31.24	533	34.19		267	34.99	333	37.97		
5 or more	92	6.14	163	10.46		107	14.02	166	18.93		
Oral contraceptive use^f^					0.12					0.16	0.93
Yes	143	9.55	124	7.95		74	9.7	68	7.75		
No	1355	90.45	1435	92.05		689	90.3	809	92.25		
Menopausal status^g^					0.01					0.01	<0.01
Pre−/Peri-menopausal	527	35.25	480	30.79		320	42.05	315	36.04		
Post-menopausal	968	64.75	1079	69.21		441	57.95	559	63.96		
Stage^h^											<0.01
In Situ	252	17.22				127	16.87				
Local	793	54.20				346	45.95				
Regional	362	24.74				249	33.07				
Distance	17	1.16				7	0.93				
Unstaged	39	2.67				24	3.19				
	**Mean**	**SD**	**Mean**	**SD**	**p value** a	**Mean**	**SD**	**Mean**	**SD**	**p value** a	**p value** b
MET hours/week^i^	24.51	29.25	24.99	29.88	0.66	21.62	31.82	20.47	29.23	0.45	<0.01
Energy intake (kcal/day)	2199.2	935.8	2097.9	888.9	<0.01	2647.2	1213.6	2540.9	1176.4	0.07	<0.01

Abbreviations: SD, standard deviation; MET, metabolic equivalent of activity; gm, grams; kcal, kilocalories.

a Case-control comparison within ethnicity using the Chi-square test for categorical variables and the *t* test for continuous variables.

b Ethnic group comparison, regardless of case-control status, using the Chi-square test for categorical variables and the *t* test for continuous variables.

c Education level. Missing data for 1 case and 1 control for non-Hispanic White women; 4 cases and 2 controls for Hispanics.

d First degree of family history. Missing data for 20 cases and 24 controls for non-Hispanic White women; 14 cases and 19 controls for Hispanics.

e Cigarette smoking status. Missing data for 1 case and 4 controls for non-Hispanic White women; 2 controls for Hispanics.

f Oral contraceptive use within five years prior to referent year.

g Menopausal status. Missing data for 3 cases for non-Hispanic White women; 2 cases and 3 controls for Hispanics.

h Stage of tumor. Missing data for 34 for non-Hispanic White women; 12 for Hispanics.

i Moderate/vigorous physical activity at referent year.

Sixty three percent of participants reported supplement intake during the referent year (71% NHW, 29% H). Intake did not differ by case-control status within each ethnic group (NHW: 47% cases vs. 53% controls; Hispanic: 45% cases vs. 55% controls, *P* = 0.25). Hispanic and non-Hispanic white women differed significantly for most nutrients (Table 2). Although Hispanic women reported higher dietary folate and vitamin B intakes than non-Hispanic white women, these differences were reversed when supplements were included. There were significant differences between Hispanic cases and controls for B_2_ and B_12_, but these differences disappeared with the inclusion of supplement intake. Among non-Hispanic white women, folate intake was significantly higher for cases at only supplemented pre-fortified levels, and intake of vitamins B_2_, B_6_ and B_12_ was higher among controls compared to cases when total intake was considered (Table 2).

**Table 2:** Mean dietary intake and total intake^a^ of folate, vitamin B_2_, vitamin B_6_, vitamin B_12_ and methionine for cases and controls, stratified by ethnicity, 4-Corners Breast Cancer Study, 1999–2004.

	non-Hispanic White					Hispanic					
	Case		Control			Case		Control			
	(*n* = 1,497)		(*n* = 1,559)			(*n* = 765)		(*n* = 877)			
	Mean	SD	Mean	SD	p valueb,c	Mean	SD	Mean	SD	p value b,c	p value d,c
Folate, prefortification (mcg)	385.25	174.86	369.79	174.25	0.260	470.30	239.39	456.05	244.54	0.842	0.073
Total DFE (mcg)^e^	767.83	1373.37	753.10	471.10	0.054	709.44	384.94	730.64	503.30	0.474	<0.001
Folate, postfortification (mcg)	415.58	182.32	397.79	182.29	0.139	541.36	269.91	519.33	268.78	0.233	<0.001
Total DFE (mcg)	798.16	1373.57	781.28	471.50	0.062	780.51	394.97	793.92	511.79	0.835	<0.001
Vitamin B_2_ (mg)	2.16	0.99	2.05	0.88	0.232	2.39	1.12	2.25	1.05	0.022	<0.001
Total (mg)	4.75	4.97	5.07	4.95	0.001	4.26	3.60	4.25	3.95	0.799	<0.001
Vitamin B_6_ (mg)	2.15	0.90	2.05	0.87	0.090	2.57	1.20	2.45	1.14	0.164	0.729
Total (mg)	5.94	12.67	6.03	11.79	0.046	4.62	5.74	5.91	32.68	0.727	<0.001
Vitamin B_12_ (mcg)	4.84	2.86	4.64	2.65	0.352	5.85	4.34	5.26	3.79	0.027	<0.001
Total (mg)	36.07	151.22	38.92	131.08	0.011	32.42	109.16	38.48	149.64	0.919	<0.001
Methionine (g)^f^	1.93	0.86	1.87	0.84	0.585	2.24	1.10	2.14	1.03	0.517	<0.001

Abbreviations: DFE, dietary folate equivalents; SD, standard deviation; mg, milligrams; mcg, microgram; g, gram.

a Dietary nutrient plus supplement. Supplement use based on participant report of multivitamins and minerals or other supplements.

b Case-control comparison within ethnicity.

c Significance test for differences between mean intakes adjusting for age, center, and total energy intake/day on logarithm transferred data.

d Ethnic group comparison, regardless of case-control status.

e Highlighted rows include dietary nutrient plus supplement.

f Supplementation not applicable.

Odds ratios for the highest quartile of folate were less than 1.0, although not statistically significant (model 1, OR = 0.84; 95%CI: 0.61, 1.15; model 2, OR = 0.90; 95%CI: 0.64, 1.26) (Table 3). Vitamin B_12_ intake was associated inversely with breast cancer risk also (model 2, OR = 0.73; 95%CI: 0.53, 1.00). Results for vitamins B_2_, B_6_, and methionine were non-significant. No statistically significant interactions were observed between folate and alcohol consumption (*P* = 0.30), menopausal status (*P* = 0.30) or ethnicity (*P* = 0.08). We also analyzed the joint effect of folate by vitamin B12. Each variable was categorized as low (Q1), moderate (Q2–Q3) and high (Q4) based on quartile distributions. The results showed that moderate folate-high vitamin B_12_ intake (OR = 0.71; 95%CI: 0.54, 0.92) and high folate-high vitamin B_12_ intake (OR = 0.76; 95%CI: 0.61, 0.93) had significant protective associations with breast cancer risk compared to low folate-low vitamin B_12_ intake. All other results were non-significant (data not shown).

**Table 3:** Multivariate-adjusted associations of selected energy-adjusted total nutrients with risk of breast cancer, 4-Corners Breast Cancer Study, 1999–2004.^a^

			Model 1^b^			Model 2^c^		
Nutrient	Quartile Intake^d^	Case No.	OR	95% CI		OR	95% CI	
Folate (mcg)^e^	≤405	598	1.00			1.00		
	405–702	605	0.94	0.75	1.18	0.98	0.77	1.23
	702–893	568	0.88	0.66	1.17	0.90	0.67	1.23
	>893	491	0.84	0.61	1.15	0.90	0.64	1.26
	*p trend*		0.45			0.77		
								
Vitamin B_2_ (mg)	≤2.16	585	1.00			1.00		
	2.16–3.42	594	1.16	0.93	1.45	1.07	0.85	1.34
	3.42–4.33	605	1.3	0.95	1.76	1.18	0.86	1.63
	>4.33	478	1.12	0.78	1.6	1.03	0.71	1.49
	*p trend*		0.4			0.72		
								
Vitamin B_6_ (mg)	≤2.29	584	1.00			1.00		
	2.29–3.87	614	1.12	0.89	1.42	1.10	0.87	1.40
	3.87–4.90	575	1.13	0.82	1.54	1.11	0.81	1.54
	>4.90	489	1.14	0.8	1.65	1.09	0.75	1.58
	*p trend*		0.65			0.80		
								
Vitamin B_12_ (mcg)	≤5.32	642	1.00			1.00		
	5.32–9.78	552	0.75	0.61	0.93	0.75	0.60	0.93
	>9.78–13.98	595	0.82	0.62	1.09	0.83	0.62	1.11
	>13.98	473	0.72	0.53	0.98	0.73	0.53	1.00
	*p trend*		0.05			0.06		
								
Methionine (g)	≤1.56	555	1.00			1.00		
	1.56–1.82	596	1.03	0.88	1.22	1.05	0.88	1.24
	1.82–2.10	559	0.99	0.83	1.17	1.00	0.84	1.18
	>2.10	552	0.99	0.83	1.17	0.98	0.82	1.17
	*p trend*		0.82			0.76		

Abbreviations: OR, odds ratio; CI, confidence interval; No., number; mg, milligrams; mcg, microgram; g, gram,

a The number of controls for each quartile is 609.

b Models adjusted for age (continuous), center, and all variables shown in table.

c In addition to the variables in model 1, models are further adjusted for ethnicity, education, body mass index, total MET hours per week, total energy intake per day, total daily fiber intake, cigarette status, alcohol intake, parity, family history, oral contraceptive use and menopausal status.

d Some values were overlapped for presentation in table due to rounding.

e Folate levels based on pre-fortification.

Total folate intake was marginally inversely associated with ER− breast cancer (OR = 0.50; 95%CI: 0.25, 1.00, *P*_trend_ = 0.07), particularly in post-menopausal women (OR = 0.28; 95%CI: 0.11, 0.71, *P*_trend_ = 0.01), while there was no association with ER+ cancer or in pre-menopausal women (Table 4). The protective effect of folate intake with ER− breast cancer was not modified by stage of breast cancer. There were no ethnic differences in these associations (data not shown). In contrast, the highest quartile of B_12_ had an inverse association with ER+ tumors (OR = 0.67; 95%CI: 0.46, 0.99, *P*_trend_ = 0.06) (Table 4), particularly in NHW women (OR = 0.49; 95%CI: 0.29, 0.81, *P*_trend_ = 0.01) and among invasive breast cancer women (OR = 0.63; 95%CI: 0.42, 0.93, *P*_trend_ = 0.01) (data not shown). Methionine was also inversely associated with risk for ER+ tumors (OR = 0.83; 95%CI: 0.66, 1.03; *P*_trend_ = 0.04), particularly in Hispanic women (OR = 0.71; 95%CI: 0.47, 1.06, *P* for trend = 0.02) (data not shown), but not with ER− cancer. The association was not modified by stage of cancer.

**Table 4:** Multivariate adjusted a,b associations of selected energy-adjusted nutrients with risk of breast cancer estrogen receptor status, 4-Corners Women’s Health Study, 1999–2004.c,d

		ER−				ER+			
Nutrient Intake	Quartile^e^	Case No.	OR	95% CI		Case No.	OR	95% CI	
Folate (mcg)^f^	≤405	99	1.00			302	1.00		
	405–702	112	0.94	0.61	1.45	304	1.02	0.77	1.36
	702–893	75	0.67	0.37	1.22	290	1.00	0.68	1.45
	>893	44	0.50	0.25	1.00	254	1.01	0.67	1.54
	*p trend*		0.07				0.92		
Vitamin B_2_ (mg)	≤2.16	94	1.00			301	1.00		
	2.16–3.42	102	1.08	0.70	1.65	296	1.01	0.76	1.35
	3.42–4.33	84	1.22	0.66	2.25	296	1.01	0.68	1.50
	>4.33	50	0.83	0.39	1.76	257	1.05	0.67	1.66
	*p trend*		0.82				0.65		
Vitamin B_6_ (mg)	≤2.29	90	1.00			297	1.00		
	2.29–3.87	113	1.42	0.92	2.19	308	1.15	0.86	1.55
	3.87–4.90	75	1.51	0.82	2.77	290	1.14	0.77	1.69
	>4.90	52	1.60	0.78	3.29	255	1.10	0.69	1.73
	*p trend*		0.23				0.76		
Vitamin B_12_ (mcg)	≤5.32	110	1.00			329	1.00		
	5.32–9.78	86	0.59	0.39	0.88	268	0.72	0.55	0.95
	>9.78–13.98	81	0.74	0.42	1.31	313	0.87	0.60	1.25
	>13.98	53	0.69	0.37	1.28	240	0.67	0.46	0.99
	*p trend*		0.29				0.06		
Methionine (g)	≤1.56	65	1.00			301	1.00		
	1.56–1.82	78	1.11	0.77	1.59	319	1.05	0.86	1.29
	1.82–2.10	96	1.37	0.96	1.96	273	0.89	0.72	1.10
	>2.10	91	1.32	0.91	1.92	257	0.83	0.66	1.03
	*p trend*		0.09				0.04		

Abbreviations: OR, odds ratio; CI, confidence interval; No., number; mg, milligrams; mcg, microgram; g, gram.

a Based on multinomial logistic regression models using controls as referent.

b Models were adjusted for all variables shown in table and for: center, ethnicity, age (continuous), education, body mass index, total MET hours per week, total calorie intake per day, total fiber intake per day, cigarette smoking, alcohol intake, parity, family history, oral contraceptive use, and menopausal status.

c Control sample size for each quartile = 609.

d Cases include *in situ* and invasive.

e Some values were overlapped for presentation in table due to rounding.

f Folate levels based on pre-fortification.

Because alcohol intake can influence the biological effects of folate and Methionine [55], we also stratified analyses by low/high alcohol consumption for all women combined and by tumor phenotype (ER−, ER+). A protective association was observed for risk of breast cancer (OR = 0.53; 95%CI: 0.37, 0.73) among women diagnosed with an ER− tumor who reported a low alcohol intake (<10 g/day) and higher folate intake (>893 mcg/day). There was no suggestion of an interaction between folate and alcohol intake among women diagnosed with ER+ tumors.

## Discussion

This case-control study provides weak evidence that dietary folate and vitamin B intake is protective for breast cancer. Several other case-control studies also have reported protective associations [8], [56], [57], but findings from cohort studies are less consistent [9], [15], [16], [58]. We also detected a protective association for vitamin B_12_, in keeping with two previous case-control studies [59], [60]. Our results, however, suggest that these associations may differ according to ER phenotype. Thus, the confounding between folate and vitamin B_12_ intake for total breast cancer risk maybe due to differential effects on ER phenotypes. We also found a protective association of methionine intake for ER+ breast cancer (*P* for trend = 0.04), but no evidence for associations with vitamins B_2_ or B_6_, in keeping with previous studies [8], [61].

A few studies to date have examined associations of dietary folate and B-vitamins with breast cancer according to ER status [23], [24], [58], [61]. Our study suggests that folate intake is marginally protective for ER− breast cancer, which is consistent with results for the Nurses’ Health Study [14] and the VITamin D and OmegA-3 TriaL (VITAL) study [10]. However, the IOWA Women’s Health Study did not find any relationship between folate intake and ER− or ER+ breast cancer [23]. The association of folate with ER− cancer is consistent with the hypothesis that low levels of folate result in global DNA hypomethylation with aberrant methylation of CpG island promotors for the ER gene, resulting in silencing of receptor expression [22], [24]. The protective associations for B_12_ and methionine with ER+ breast cancer agree with the hypothesized role of one-carbon metabolism in carcinogenesis but suggest a different pathway [62]. Vitamin B_12_ and methionine are established enzymatic cofactors in one-carbon metabolism [4], [63]. Deficiencies in these vitamins dysregulate one-carbon metabolism and impair DNA synthesis and repair, which may increase the risk of carcinogenesis [64]. Taken together, our results suggest that folate deficiency may be more important than B_12_ or methionine for alteration of DNA methylation, resulting in silencing of ER expression and the manifestation of the ER− breast cancer phenotype. In contrast, deficiency of B_12_ and methionine may influence the risk for the more common ER+ phenotype by a more general impairment of DNA synthesis and repair (29).

We used pre-fortification values for folate intake in our study because this was the relevant exposure before the introduction of fortification. In more recent studies conducted post-fortification, the protective association of increased folate intake on breast cancer risk may be nullified or even reversed [16], [65], [66]. Choumenkovitch et al. [67] estimated that the 1998 folate fortification increased average folate intake for women in the U.S. by approximately 100 mcg/day. As a result, compared to the recommended dietary allowance of 400 mcg/day of dietary folate equivalents (DFE) for adults [68], the current folate intake of the U.S. population may be more than adequate to saturate metabolic systems: 665 mcg DFE/day on average for women without supplemental intake and 1,013 mcg DFE/day for women with supplemental intake [67]. DFE’s are commonly used folate intake units calculated from natural folate from food and synthetic folic acid from dietary supplements or fortified foods. [69] A recent report from the Prostate, Lung, Colorectal, and Ovarian Cancer Screening Trial (PLOC) suggests that excess folate intake due to fortification and supplementation may actually increase breast cancer risk in post-menopausal women [70]. Animal studies also provide evidence for an increased risk with excessive intake, with timing and dose of exposure being the key factors [66]. Thus, the timing and dose-effect relationship of folate with breast cancer risk may actually be U-shaped [1]. The levels at which risk decreases or increases remain to be determined, and could be highly variable depending upon diet composition and genetic susceptibility. In our study, mean total folate intake was similar to that reported for the PLOC study, but lower than the levels reported by Sellers et al. [23], [24] and Zhang et al. [23], [24]. Additionally, the difference between pre- and post-fortification intake for women in our study was smaller than the average increase reported by Choumenkovitch et al. [67]. Women living in the 4-Corners area of the Southwest, particularly Hispanics, appear to have a higher intake of folate from diet, but a lower intake of folic acid from fortification and supplementation, resulting in an overall lower folate status compared with the general US population. It is therefore possible that the protective association of folate on breast cancer is less masked and especially not reversed in our study by folate supplementation and fortification.

The strengths of this study include a large population-based study sample including Hispanic and non-Hispanic white women with complete and extensive data for diet, dietary supplements, and numerous non-dietary confounding factors, collected with rigorous quality-control standards [50]. Inclusion of vitamins B2, B6, and B12 as covariates in the analyses helped to clarify the association between these vitamins in relation to breast cancer risk. Additionally, cases were identified through population-based cancer registries.

Several limitations must be considered. We were unable to account for genetic variants that regulate one-carbon metabolism and that have been shown to be associated with increased breast cancer risk [71], [72]. Several studies have reported that women with a low folate intake and the MTHFR 677TT genotype are at increased risk of breast cancer [57], [73], [74], although Lewis et al.’s [75] meta-analysis produced a significant summary estimate for MTHFR677. As in all case-control studies using self-report methods, recall bias is possible. Twenty-four percent of cases with invasive cancer were missing ER status, however, this is not out of keeping with other studies utilizing ER status [76]. Additionally, there was little difference for missing ER status between Hispanic (24.7%) and non-Hispanic white (23.0%) women diagnosed with invasive cancer (*P* = 0.41), and overall, it is unlikely that missing ER status is associated with folate intake. The participation rate was lower in our study than for some previous studies, increasing the potential for selection bias, although obtaining high response rates in minority populations is a universal problem [77], [78]. To obtain a better understanding of the potential impact of poor response on study findings, we evaluated several factors and showed comparability for participants and non-participants [28]. Also, Larsson et al. [8] found no association across studies between response rates and risk estimates for folate and breast cancer in their meta-analysis. Lastly, the sample size was not sufficient to detect weak associations. Nonetheless, our study provides a unique insight into the association of folate and vitamin B intake on breast cancer risk in Hispanic women.

In conclusion, our results support the hypothesis that folate and B-vitamin intake influence breast cancer risk, but that folate intake may be related specifically to risk for the ER− phenotype. These risks may be different than those reported in studies conducted after folate fortification.
